# Health Management and Evidence-Based Medicine

**DOI:** 10.1590/S1679-45082013000300002

**Published:** 2013

**Authors:** Marcia Makdisse, Marcelo Katz

**Affiliations:** 1 Hospital Israelita Albert Einstein, São Paulo, SP, Brazil.

Health management involves a variety of activities that include daily decisionmaking. Two types of decisions deserve attention: one that enables the manager to choose a specific direction (scenario A) and another that enables the manager to act and sometimes correct existing pathways or processes in order to improve quality of care (scenario B).

When a specific direction (scenario A) is chosen, it is prioritized in relation to the other possibilities. In this context, modern concepts of health economics may help in choosing the best option. Health economics states that available resources must be deployed efficiently. Efficient allocation of resources involves not only monetary but also scientific issues. The goal should be to choose the practice that is both clinically and cost-effective. In regards to scenario B, quality tools and concepts should be applied to guarantee the uniformity and high performance of processes (“doing things right”).

How could evidence-based medicine (EBM) help clinicians in the decisionmaking process? EBM, which gained strength in the 1990s, seeks to integrate the best scientific evidence available into daily clinical practice. In patient care, this can be expressed as “doing the right things.” However, defining the right things is a hard task given the huge amount of information available. The number of published papers in electronic databases grows progressively and continuously. For this reason, being aware of scientific methodology can help clinicians interpret and select high quality studies that are more applicable to their practice. In **scenario A**, EBM offers scientific background and points out more effective practices. It also helps health care providers to evaluate the strengths and limitations of costeffectiveness studies and how to apply the results to real-world health services. In **scenario B**, EBM merges with clinical quality improvement tools to create the concept of Evidence-based quality improvement (“doing the right things right”)^(1)^. When health care providers do the right things right, quality of care and clinical outcomes are improved. The cycle is completed by continuous performance measurement, adjusting actions according to the best evidence available at a given moment. This synergy between management and EBM could be described as “doing the right things even better.”

In cardiology, a vast amount of scientific evidence is available. For example, a quick search via PubMed using the Medical Subject Headings term cardiology retrieves about 163,000 articles. As a consequence, one should expect a similar availability of studies focused on quality improvement that could benefit patients, also considering physician experience and patient expectations.

Through initiatives from the Institute of Healthcare Improvement and national and international Cardiology Societies, as well as performance indicators demanded by hospital accreditation agencies (mainly on acute myocardial infarction and heart failure), health services have created a support structure to monitor practice and design projects for continuous quality improvement^(2,3)^. However, few studies on this subject have been published so far, especially on a national basis.

The article “Effect of implementing an acute myocardial infarction guideline on quality indicators”^(4)^ in this issue shows several challenges and actions implemented at a private hospital with a mixed clinical staff during an 8-year follow-up. It emphasizes the role of management and care leadership commitment, clinical staff engagement, organizational culture, knowledge of care process, and continuous monitoring of indicators. This current theme issue on cardiology at **einstein** features articles that address a wide variety of topics in cardiology and bring together information that is both relevant and of high scientific quality. We hope that you benefit from this issue. Enjoy your reading.
